# From cervical cancer elimination to eradication of vaccine-type human papillomavirus: Feasibility, public health strategies and cost-effectiveness

**DOI:** 10.1016/j.ypmed.2020.106354

**Published:** 2021-03

**Authors:** Mark Jit, Kiesha Prem, Elodie Benard, Marc Brisson

**Affiliations:** a Department of Infectious Disease Epidemiology, London School of Hygiene & Tropical Medicine, Keppel Street, London WC1E 7HT, United Kingdom; b School of Public Health, University of Hong Kong, Patrick Manson Building, 7 Sassoon Road, Hong Kong SAR, China; c Saw Swee Hock School of Public Health, National University of Singapore, 12 Science Drive 2, #10-01, 117549, Singapore; d Centre de recherche du CHU de Québec - Universite Laval, Québec, QC, Canada; e Department of Social and Preventive Medicine, Universite Laval, Québec, QC, Canada; f MRC Centre for Global Infectious Disease Analysis, Department of Infectious Disease Epidemiology, Imperial College London, London, UK

**Keywords:** Human papillomavirus, Cervical cancer, Vaccination, Eradication

## Abstract

The Director-General of the World Health Organization has called for global action towards elimination of cervical cancer as a public health problem. Cervical cancer is caused by human papillomavirus (HPV), an infectious agent with no non-human reservoir. One way to achieve this is through very high levels of vaccine coverage that could enable global eradication of vaccine-type HPV. Using the case study of India, we show that HPV eradication can meet all the Dahlem and Strüngmann criteria for feasibility of eradication. It can be achieved with 90% gender-neutral HPV vaccine coverage together with 95% coverage in high-risk groups such as female sex workers. Such a strategy would likely be cost-effective compared to no vaccination. Although it would be more costly in the short-term than achieving cervical cancer elimination alone, it would save costs in the long-term by removing or at least sharply reducing the need for preventive measures.

## Highlights

• Eradication of vaccine-type HPV is a way to permanently maintain cervical cancer elimination.
• Eradication is technically feasible, likely cost-effective and equitable.
• Eradication requires high gender-neutral vaccine coverage, particularly in high-risk groups such as sex workers.

## 1 Introduction

Infection with an oncogenic (or high-risk) genotype of human papillomavirus (HPV) is the cause of all or almost all cases of cervical cancer, and has been linked to many cases of anal, vulvar, vaginal, penile and head and neck cancer (de Martel et al., 2017). Prophylactic vaccines are available that are safe and highly efficacious against persistent infection with high-risk HPV types (Schiller et al., 2012). Three vaccines that are widely licensed protect against HPV 16 and 18, which are linked to 70% of cervical cancer. One of them also protects against HPV 31, 33, 45, 52 and 58, which are linked to a further 20% of cervical cancers (de Martel et al., 2017). Efforts to develop vaccines with broad protection against all HPV types are in progress (Schiller and Müller, 2015). Secondary prevention of cervical cancer is possible through screening for the presence of high-risk HPV types and abnormal neoplasias that are precursors to cervical cancer.

The widespread availability of both vaccines and screening methods has the potential to bring about large reductions in cervical cancer incidence globally. Countries which achieved high coverage in population-based HPV vaccination programmes reported 85% reductions in HPV 16/18 among 15–19 year old females 5–8 years after programme initiation, with large reductions also reported in males and older females due to herd effects (Drolet et al., 2019). In practice, impact is limited because uptake of both prevention methods is limited in less developed countries that account for 70% of cervical cancer (de Martel et al., 2017). To accelerate roll-out, the Director-General of the World Health Organization has called for global action towards elimination of cervical cancer as a public health problem. The strategy involves achieving 90% coverage of HPV vaccination in women, 70% coverage for screening and 90% coverage for treatment of cervical lesions and cancers (the “90–70-90 strategy”) (Canfell et al., 2020).

Cervical cancer is caused by an infectious agent with no non-human reservoir (Campo, 2003). Since HPV vaccines have very high efficacy against persistent vaccine-type HPV infection (Schiller et al., 2012), very high levels of vaccine coverage would eradicate HPV types causing almost all cervical cancer cases; unlike elimination it would also remove or at least sharply reduce the need for preventive measures in future. Global coordination to achieve very high coverage combined with herd immunity has led to eradication of smallpox, reduction of poliomyelitis and dracunculiasis to a handful of cases annually, and elimination of measles and rubella in many countries (Roser et al., 2014). This demonstrates the technical feasibility of eradication. However, unlike HPV, none of these pathogens are sexually transmitted. Hence, the feasibility, methodology and requirements of an HPV eradication effort needs to be assessed using an approach specific to the characteristics of this virus.

This article discusses the feasibility, public health and economic case for eradication of vaccine-type HPV. First, we define eradication and examine the technical feasibility of HPV eradication according to widely accepted criteria. Then we use epidemiological and economic modelling to explore key criteria around the public health strategies needed to achieve eradication, and the cost-effectiveness of these strategies, using India as a case study.

## 2 Definition of elimination and eradication

According to the WHO's generic framework for control, elimination and eradication of neglected tropical diseases, a pathogen is eradicated when its prevalence is reduced to zero, as a result of deliberate efforts, with no more risk of reintroduction (World Health Organization, 2016). By that definition, the only human pathogen to be eradicated is smallpox. A step towards eradication is elimination, which is defined as “elimination of transmission to zero infection incidence by a specific pathogen in a defined geographical area, with minimal risk of reintroduction, as a result of deliberate efforts” (World Health Organization, 2016). Hence a pathogen is eradicated when it is eliminated in all locations. Both are distinguished from elimination as a public health problem, defined as the achievement of measurable global targets set by WHO in relation to a specific disease, as a result of deliberate efforts (World Health Organization, 2016).

The current WHO elimination initiative aims to bring cervical cancer incidence to below 4 cases per 100,000 women in every country (Brisson et al., 2020a). This will satisfy the definition of elimination of cervical cancer as a public health problem, but it will not necessarily lead to HPV elimination in any region, nor to global eradication, since HPV can still be circulating even when this target is reached.

## 3 Is eradication technically and practicably feasible?

The world has seen a number of infectious disease eradication efforts, beginning with efforts to eliminate yellow fever from the Western Hemisphere in the early 1900s (Aylward et al., 1998). In 1997, the Dahlem Workshop on the Eradication of Infectious Diseases was established to establish the technical and socioeconomic criteria for a successful eradication effort (Dowdle and Hopkins, 1998). These were reaffirmed and expanded in the 2010 Ernst Strüngmann Forum on Disease Eradication in the Context of Global Health in the 21st Century (Strebel et al., 2011). Table 1 summarises the key criteria established, as well as the extent to which vaccine-type HPV satisfies them. All the criteria for eradication are clearly met with the exception of the need to establish an eradication strategy, economic arguments and financial feasibility, where further epidemiological and economic modelling is needed.

**Table 1:** Summary of Dahlem (Dowdle and Hopkins, 1998) and Strüngmann (Strebel et al., 2011) criteria for a successful effort to eradicate and infectious disease, and the extent to which they are satisfied by vaccine-type HPV.

Criteria for successful eradication	Status for vaccine-type HPV
Humans are essential to the life cycle of the pathogen	Humans are the only natural reservoir for HPV (Centers for Disease Control and Prevention, 2015).
Effective intervention available to interrupt transmission	HPV vaccines have high prophylactic efficacy against persistent infection by vaccine-type HPV (Schiller et al., 2012).
Practical diagnostic tools with high sensitivity and specificity to infection	HPV DNA tests have very high sensitivity to both HPV infection and disease, and very high specificity to HPV infection (Mustafa et al., 2016).
Intervention is safe, cheap and can be scaled-up to national level	HPV vaccines have excellent safety profiles (Schiller et al., 2012). Demonstration projects in low- and middle-income countries have achieved high coverage (Gallagher et al., 2017).
Political and social commitment at the highest level	The Director-General of the WHO has called for cervical cancer elimination. The strategy was adopted by the World Health Assembly in 2020 (World Health Assembly, 2020).
Technically feasible eradication strategy	Needs to be established through epidemiological modelling of options (see below)
Strong economic arguments	Economic modelling needed (see below)
Strong ethical arguments	Meets all ethical criteria of duty to rescue, duty to future generations, equity and contribution to global public good.
Financial feasibility	Economic modelling needed (see below)

## 4 Is there a technically feasible eradication strategy?

Since vaccinating every individual in the world is infeasible, eradication efforts have relied on sustained high vaccine coverage providing herd protection for the vast majority of the population, combined with surveillance and special vaccination programmes to close immunity gaps and contain outbreaks (Klepac et al., 2013; Fenner et al., 1988). None of the pathogens currently targeted for elimination are sexually transmitted, so HPV eradication will have its own specificities. In particular, herd effects will depend on sexual behaviour which differs widely across the world. In many countries a high proportion of sexually transmitted infections occur due to transmission from core groups (such as sex workers) with much more frequent sexual contacts than non-core groups (Wasserheit and Aral, 1996).

Epidemiological modelling suggests that achieving 90% female-only vaccination coverage will dramatically reduce cervical cancer incidence in every country (Brisson et al., 2020a). However, it will not bring cervical cancer incidence below 4 cases per 100,000 women in every country as some countries in sub-Saharan Africa will still have cervical cancer incidence above the elimination as a public health problem threshold. However, combining 90% female-only vaccination coverage with 70% screening coverage (as in the 90–70-90 strategy) can bring cervical cancer incidence below this threshold.

Even achieving this threshold will not eradicate vaccine-type HPV, particularly not HPV 16 which is responsible for more cancers than any other HPV type. A meta-analysis of predictions from 16 transmission dynamic models of HPV vaccination in high-income countries demonstrated that 90% female-only coverage is insufficient to eliminate HPV 16 transmission, although 90% coverage in both males and females (i.e. gender-neutral) may be sufficient (Brisson et al., 2016). Since HPV 16 requires the highest vaccine coverage to eliminate (Baussano et al., 2017), other vaccine types are likely to have already been eliminated when HPV 16 elimination is achieved.

**Fig. 1:**
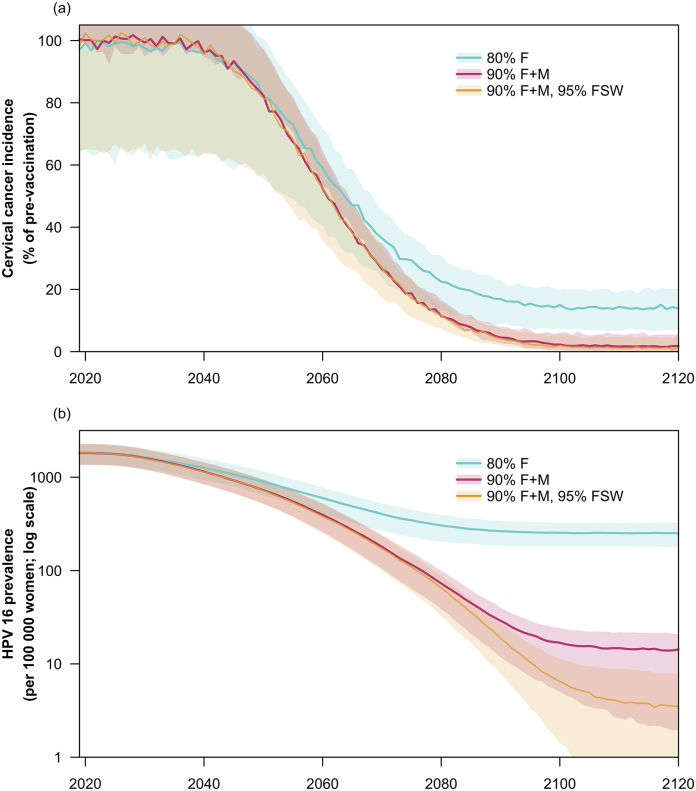
Long-term impact on cervical cancer and on HPV 16 prevalence of 3 vaccination strategies: 80% coverage for vaccination of 10-year females (80% F), 90% coverage for vaccination of all 10-year olds (90% F + M) and 90% coverage for vaccination of all 10-year olds together with 95% coverage for vaccination of female sex workers (90% F + M, 95% FSW). Both outcomes are standardised according to the WHO world standard population.

The meta-analysis did not look at model predictions for low- and middle-income countries. Hence we examined different scenarios about HPV vaccination in India from HPV-ADVISE (Brisson et al., 2020b), an individual-based dynamic model of HPV transmission and natural history (see Appendix for details). Fig. 1 shows the results, suggesting that 90% coverage in both sexes will bring HPV 16 prevalence close to elimination. Here we define viral elimination as reducing prevalence to below 10 per 100,000 in the population. At this level, stochastic extinction is possible as long as the probability of importation from other populations is small (a condition which would hold if the world was aiming for HPV eradication) (Craig et al., 2015). However, even at this coverage level, HPV transmission can be sustained in a small group of sex workers and their clients. Additionally achieving 95% coverage in 10-year old girls who become female sex workers (e.g. through targeted outreach to vulnerable populations) will likely achieve the goal of elimination.

These levels of coverage are challenging but not impossible to reach. Demonstration projects in low- and middle-income countries often reach or exceed 90% coverage (Gallagher et al., 2017). Out-of-school rates for lower secondary school age in 153 countries reporting recent (2013–2018) data to UNESCO are less than 5% in 48% of countries, and less than 15% in 72% (UNESCO Institute for Statistics, 2020). A combination of school, health facility and outreach-based strategies may be able to achieve the highest levels of coverage. In addition, outreach programmes to screen, treat and vaccinate sex workers could ensure that this at-risk group are cleared of HPV infections and protected from subsequent infections.

## 5 Are the costs of eradication worth the benefits?

HPV vaccination of females at 80% coverage prior to sexual debut has been shown to be highly cost-effective globally (Jit et al., 2014; Abbas et al., 2020). These cost-effectiveness analyses assume costs for vaccine delivery from HPV demonstration projects that have mostly been able to achieve these levels of coverage.

Eradicating vaccine-type HPV would likely have much higher costs for two reasons. Firstly, the targeted population would double since males would need to be vaccinated also. Secondly, the cost per person vaccinated would increase sharply in the move from 80% to 90% coverage since the remaining unvaccinated population will be harder to reach. Studies have not looked at the cost of scale-up to this level of coverage for HPV, but for measles an assessment has been made of costs of scale-up in hard-to-reach populations to reach 90% population coverage based on interviews with health officials (Bishai et al., 2010). This estimated costs per vaccinated child of $37.93 in Bangladesh, $27.40 in Ethiopia and £35.83 in Uganda (compared to about $1 per child prior to scale up). These higher costs funded travel by vaccinators to remote areas, vaccine demand creation activities as well as registration systems to identify unvaccinated individuals.

These higher costs may be justified because eradicating vaccine-type HPV would have larger long-term benefits than eliminating cervical cancer as a public health problem without eradicating the underlying infectious agent. In principle, eradication should allow large cost savings from the cessation of cervical cancer preventive activities (vaccination and screening), since there will no longer be a virus to protect against - the so-called “eradication dividend” (Barrett, 2013). This is well illustrated by the case of smallpox. Smallpox eradication cost about USD 300 million but saves the world over a billion USD a year (in 1967 USD) (Fenner et al., 1988) - these benefits are still being realised even though the costs of eradication today are minimal (since we no longer need to vaccinate against it).

To illustrate, we calculated total costs and cost-effectiveness in order to reach elimination of HPV 16 infection in India, assuming that eradication will then be achieved globally in the year it is reached in India, after which most vaccination activities can be ceased. We used epidemiological outcomes from HPV-ADVISE (Brisson, 2020) and economic parameters from PRIME (Jit et al., 2014; Abbas et al., 2020). Detailed assumptions are given in the Appendix.

**Fig. 2:**
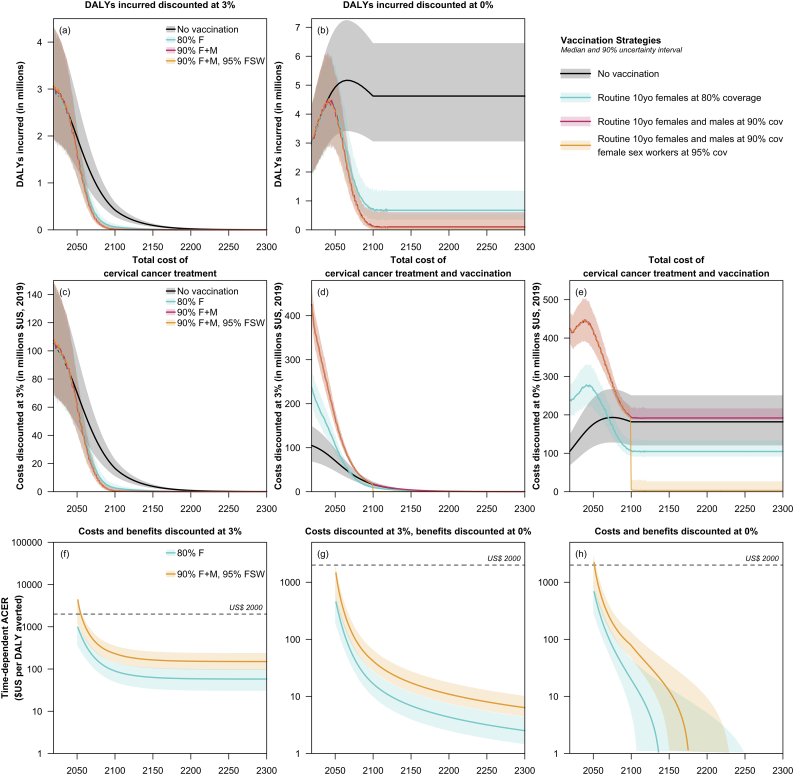
Disability-adjusted life-years (DALYs) incurred, cervical cancer treatment and vaccination costs, and time-dependent average cost-effectiveness ratios (ACERs) for all vaccination strategies. Both DALYs and costs were discounted at 0% and 3%. The ACER for two strategies—80% coverage for vaccination of 10-year females (80% F) and 90% coverage for vaccination of all 10-year olds together with 95% coverage for vaccination of female sex workers (90% F + M, 95% FSW)— was calculated compared to the no vaccination scenario. It is calculated based on totals from 2019 to the year on the x-axis. In 2019, India's GDP per capita is approximately US$2000. All costs are presented in 2019 US$.

**Fig. 3:**
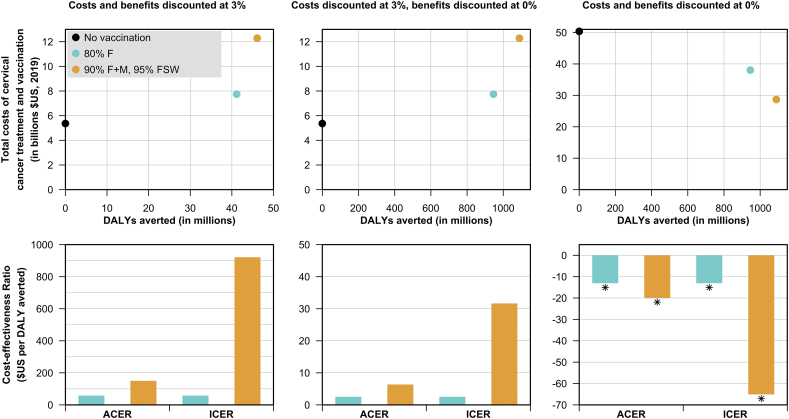
Net costs, DALYs incurred, average (i.e. vs no vaccination) and incremental cost-effectiveness ratio (i.e. vs the next most costly option) for different vaccine options and discounting regimes. All outcomes are totalled over the years 2019–2300. ACER: average cost-effectiveness ratio, ICER: incremental cost-effectiveness ratio. Starred (*) bars indicate cost saving strategies.

Fig. 2 shows the net costs, DALYs incurred and average cost-effectiveness ratio of the three strategies compared to no vaccination. Fig. 3 shows both average (i.e. vs no vaccination) and incremental (i.e. vs the next most costly option) cost-effectiveness ratio. An eradication strategy will cost more at first, due to the need to deliver more than twice as many doses, but after 2100 will allow vaccination costs to drop to zero. Indeed the cost savings of eradication are underestimated because they do not take account increases in the cost of cervical screening as part of WHO's 90–70-90 strategy. If next generation vaccines preventing all or almost all high-risk HPV types are deployed for eradication strategies, then screening costs will also eventually reduce to zero. Both an elimination strategy (80% female-only vaccine coverage) and an eradication strategy (90% gender-neutral vaccine coverage, with 95% coverage in female sex workers) are likely to be cost-effective compared to India's GDP per capita threshold. If benefits are undiscounted, both strategies could be cost-effective even with a much lower threshold below $50 per DALY averted.

## 6 Discussion

The WHO Director-General's call for action on elimination of cervical cancer as a public health issue is an important and necessary stimulus for more widespread introduction of the tools we already have for preventing cervical cancer, particularly in the regions where cervical cancer burden is highest. However, this article argues that in the long run it is insufficiently ambitious. Eradication of vaccine-type HPV itself is feasible, cost-effective (compared to no vaccination) and will eventually bring greater and more equitable benefits. An eradication objective is not incompatible with elimination as a public health problem - indeed, elimination is a necessary step on the road to eradication. However, elimination is largely a national public good. Eradication is a global public good so there is a much stronger case for international cooperation and resource pooling to achieve it, as was seen in the smallpox and polio eradication initiatives.

The distinction between elimination as a public health problem and eradication may seem academic, but it will become increasingly important as we approach the disease end game. Indeed, the term “elimination as a public health problem” may eventually become a counter-productive term if it gives the impression that cervical cancer is a solved problem even though the underlying pathogen continues to circulate. Leprosy offers an important lesson; interest in leprosy research has diminished following declaration of its elimination as a public health problem even though cases continue to occur (Fine, 2007). Even at the early stages, an eventual eradication goal can spur consideration of more equitable strategies. Gender-neutral vaccination and consideration of vulnerable groups such as sex workers, men who have sex with men and people in geographically remote areas are crucial for eradication. However, the high cost of reaching these groups can cause them to be overlooked when seeking cervical cancer elimination without HPV eradication. Indeed, men who have sex with men have a high HPV-related disease burden but derive very little benefit from a cervical cancer elimination goal focused on female vaccination and screening only. However, they are a crucial part of an eradication goal.

Given the higher costs and number of doses needed to achieve eradication, countries may wish to aim for elimination targets first (i.e. attain 80% female-only vaccine coverage as the initial goal) but then switch to a more equitable eradication strategy as soon as local resources and global vaccine supplies permit.

Eradication of vaccine-type HPV with current vaccines will not reduce the incidence of cervical cancer to zero since 10% of cervical cancers are due to types not in any current vaccine. Hence cervical screening may still be necessary, even though the frequency of screening can likely be sharply reduced (Kim et al., 2017; Simms et al., 2016). However, in the decades it takes to eradicate vaccine-type HPV, it is likely that HPV vaccines with additional antigens or with complete and lasting cross-protective immunity will be developed (Schiller and Müller, 2015). The remaining high-risk HPV types can then be eliminated with lower coverage and over less time than it takes to eliminate HPV 16 (Baussano et al., 2017). This would mean that cervical cancer would become a disease that is fully or almost fully vaccine-preventable, and hence eradicable.

The benefits of eradication will take many decades before they are realised. Unlike other infections targeted for eradication in the past (such as smallpox and polio), HPV can persist in its host for decades. Treatment of cervical lesions clears the infection in some but not all individuals (Cuschieri et al., 2016). However, the timescale for elimination of cervical cancer as a public health problem is also measured in decades (Brisson et al., 2020a), yet this has not deterred the global political will to achieve this target. Achievement of elimination as a public health problem can be regarded as a milestone towards the greater prize of eradication.

## Funding statement

MJ and KP were funded by the 10.13039/100000865Bill & Melinda Gates Foundation (OPP1157270). MJ was funded by the 10.13039/501100000272National Institute for Health Research (NIHR200929) Health Protection Research Unit in Immunisation at LSHTM in partnership with Public Health England. The views expressed are those of the author(s) and not necessarily those of the Bill & Melinda Gates Foundation, the National Health Service, the NIHR, the Department of Health and Social Services, or Public Health England.

## Declaration of Competing Interest

None declared.

## Appendix A Cost-effectiveness of strategies to eliminate cervical cancer and to eradicate vaccine-type HPV infection in India

### A.1 Vaccination strategies considered

To assess the cost-effectiveness of vaccination strategies which could lead to eradication of vaccine-type HPV, we compared the impact of three vaccination strategies: 80% coverage for vaccination of 10-year old females (80% F), 90% coverage for vaccination of all 10-year olds (90% F + M) and 90% coverage for vaccination of all 10-year olds together with 95% coverage for vaccination of female sex workers (90% F + M, 95% FSW) against the no HPV vaccination scenario.

### A.2 Modelling approach

The analyses combined epidemiological outcomes from an individual-based dynamic model, HPV-ADVISE (Brisson, 2020), and economic parameters from a multi-cohort, static, proportional impact model, Papillomavirus Rapid Interface for Modelling and Economics (PRIME) (Jit et al., 2014; Abbas et al., 2020). Stratified by age, sex and sexual activity-based risks and screening behaviour-based risks, the HPV-ADVISE model also accounts for the HPV natural history and disease, and empirically-measured HPV transmission rates. We examined outcomes for a single country (India), assuming that global eradication of vaccine-type HPV would be achieved in the same year that vaccine-type HPV elimination is achieved in India.

Female sex workers and their clients are represented in the model as a separate level of sexual activity for females and males respectively (out of the four levels for females and three levels for males). Males can act as a “bridge” population between female sex workers and their regular partners. Details of the parameterisation are shown in the HPV-ADVISE technical appendix (Brisson et al., 2020b). The data sources used for the parameters are in Table A4, and proportion of ex workers and men who visit them are in Fig. A2 (L3 sexual behaviour category). For India these are:

Proportion of women who are female sex workers: median 0.6% (10–90 percentile 0.4–0.9).

Proportion of men who visit female sex workers: median 5.7% (10–90 percentile 4.0–8.4).

### A.3 Epidemiological impact

Because the eradication of vaccine-type HPV might only be achieved after several decades, we projected the age-specific incidence and prevalence rates from HPV-ADVISE and dynamic population age composition from UN World Population Prospects (UNWPP) to the year 2300. We then estimated the numbers of cervical cancers for the years 2019 to 2300 by multiplying the projected age-specific incidence for India with India's projected female population by age for all vaccination strategies. Under the no vaccination scenario, we multiplied the age-specific incidence rate in 2019 (pre-vaccination) to the projected population age structure for 2019–2300. To determine the year eradication is achieved, we first calculated the prevalence of HPV 16/18 (genotypes covered by all licensed vaccines) for the years 2019 to 2300. We then identified the year at which the projected prevalence of HPV 16/18 falls below 10 per 100,000 in the population.

### A.4 DALYs incurred

Using the disability-adjusted life-years (DALYs) incurred and cost for cervical cancer treatment per cervical cancer case from PRIME, we estimated total DALYs incurred and total treatment costs (in 2019 USD) by scaling it by the total number of cervical cancer cases for all scenarios.

### A.5 Cost of vaccination

To derive the cost of vaccination under the three vaccination strategies, we first calculated the number of individuals vaccinated and the number of vaccine doses administered routinely and uninterrupted over the years 2019–2300. We assume that every immunised individual received two doses of the HPV vaccine, and the procurement cost per dose is USD 4.50. The vaccine delivery costs are assumed to be USD 5 per fully immunised individual for the first 90% of the target population (Levin et al., 2014). We assumed that the remaining 5% were in hard-to-reach groups, and would cost USD 30 per fully immunised individual, based on estimates of the cost of reaching hard-to-reach children with measles vaccination (Bishai et al., 2010).

The total cost of vaccination is the sum of both procurement and delivery costs. Once eradication is reached, we assumed that the cost of vaccination drops to zero.

### A.6 Cost-effectiveness

Both health effects and costs were discounted at 0% and 3%. We measured the average cost-effectiveness ratio (ACER) of the three vaccination strategies compared against the no vaccination scenario, and the incremental cost-effectiveness ratio (ICER) of the strategies compared to next most-costly strategy, by calculating the cost per DALY averted (in 2019 USD). We considered the strategy to be cost-effective if the ICER was less than the GDP per capita of India (around USD 2000 in 2019).

### A.7 Results

The eradication target for vaccine-type HPV (prevalence less than 10 per 100,000 in the population) was only reached in the third scenario (in the year 2100) where the coverage for vaccination of all 10-year olds and female sex workers were 90% and 95%, respectively. Beyond 2100, the costs of vaccination were zero for the third scenario (Fig. 2). The vaccination strategies became cost-effective after 23 years (in 2042) for the lower coverage strategy and 30 years (in 2049) for the higher coverage strategies. The cost per DALY averted levelled off at approximately US$ 58 and US$ 151 (Fig. 2) for the strategies where 80% coverage for vaccination of 10-year old females (80% F) and 90% coverage for vaccination of all 10-year olds together with 95% coverage for vaccination of female sex workers (90% F + M, 95% FSW), respectively. When health effects were undiscounted, the cost per DALY averted levelled off at much lower values at approximately US$ 3 and US$ 6 (Fig. 2), respectively.
